# An analysis of views about supported reduction or discontinuation of antipsychotic treatment among people with schizophrenia and other psychotic disorders

**DOI:** 10.1186/s12888-022-03822-5

**Published:** 2022-03-15

**Authors:** Nadia E. Crellin, Stefan Priebe, Nicola Morant, Glyn Lewis, Nick Freemantle, Sonia Johnson, Rob Horne, Vanessa Pinfold, Lyn Kent, Ruth Smith, Katherine Darton, Ruth E. Cooper, Maria Long, Jemima Thompson, Lisa Gruenwald, Robert Freudenthal, Jacki L. Stansfeld, Joanna Moncrieff

**Affiliations:** 1 Research & Development Department, Goodmayes Hospital, North East London NHS Foundation Trust, Essex, IG3 8XJ UK; 2 Division of Psychiatry, University College London, London, W1T 7NF UK; 3 Unit for Social and Community Psychiatry, Queen Mary University of London, London, E1 4NS UK; 4 Institute of Clinical Trials and Methodology, University College London, London, WC1V 6LJ UK; 5 School of Pharmacy, University College London, London, WC1N 1AX UK; 6 The McPin Foundation, London, SE1 4YR UK; 7 Independent consultant, London, UK; 8 University of Greenwich, Faculty of Education, Health and Human Sciences, London, SE10 9LS UK; 9 East London NHS Foundation Trust, Newham Centre for Mental Health, London, E13 8SP UK; 10 Faculty of Medical Sciences, University College London, London, UK; 11 Barnet Enfield Haringey Mental Health NHS Trust, London, UK

**Keywords:** Schizophrenia, Psychosis, Antipsychotics, Mixed methods

## Abstract

**Background:**

Antipsychotic medication can reduce psychotic symptoms and risk of relapse in people with schizophrenia and related disorders, but it is not always effective and adverse effects can be significant. We know little of patients’ views about continuing or discontinuing antipsychotic treatment.

**Aims:**

To explore the views of people with schizophrenia and other psychotic disorders about continuing their antipsychotic medication or attempting to reduce or discontinue this medication with clinical support.

**Methods:**

We collected quantitative and qualitative data by conducting semi-structured interviews in London, UK. Factors predicting a desire to discontinue medication were explored. Content analysis of qualitative data was undertaken.

**Results:**

We interviewed 269 participants. 33% (95% CI, 27 to 39%) were content with taking long-term antipsychotic medication. Others reported they took it reluctantly (19%), accepted it on a temporary basis (24%) or actively disliked it (18%). 31% (95% CI, 25 to 37%) said they would like to try to stop medication with professional support, and 45% (95% CI, 39 to 51%) wanted the opportunity to reduce medication. People who wanted to discontinue had more negative attitudes towards the medication but were otherwise similar to other participants. Wanting to stop or reduce medication was motivated mainly by adverse effects and health concerns. Professional support was identified as potentially helpful to achieve reduction.

**Conclusions:**

This large study reveals that patients are commonly unhappy about the idea of taking antipsychotics on a continuing or life-long basis. Professional support for people who want to try to reduce or stop medication is valued.

**Supplementary Information:**

The online version contains supplementary material available at 10.1186/s12888-022-03822-5.

## Introduction

Antipsychotic medication is considered the primary treatment for people with a diagnosis of schizophrenia and related disorders across the world [1–4]. It is effective in reducing acute symptoms [5] and long-term antipsychotic medication is recommended for people with recurrent episodes on the basis of evidence that continuous antipsychotic treatment reduces the risk of relapse compared to discontinuation [6, 7]. However, the adverse effects of these drugs are often serious and disabling, including diabetes, tardive dyskinesia, heart disease, sedation, emotional blunting, akathisia and sexual dysfunction [8–13]. In addition, the beneficial effects of long-term treatment may have been over-estimated given that most previous studies have compared maintenance antipsychotic treatment to abrupt discontinuation without controlling for the impact of withdrawal effects (which are more likely following abrupt discontinuation) [14–17].

Studies of the long-term outcomes of naturalistic, non-randomised cohorts give conflicting results about the benefits of maintenance antipsychotic treatment, with some suggesting it improves outcomes [18] and some reporting that people who avoid long-term treatment do better [19–21]. Few randomised trials have followed people up for more than a few months, and evidence from such studies suggests that the impact of discontinuation lessens over time [6]. Two randomised trials report results of a long-term follow-up involving people with a first episode of psychosis. One included people in stable remission (remission for 6 months) and found that gradual antipsychotic reduction had beneficial effects on social functioning in the long-term [22]. The other reported that a more abrupt process of discontinuation had detrimental effects [23], with early psychotic relapse linked to poorer outcomes [24]. However, a composite outcome and inclusion of some data from the end of the original trial may have influenced results, and no difference was observed in symptom levels or functioning [25]. Several authors have since discussed reasons for the uncertainty of current evidence on the implications of antipsychotic discontinuation, including a lack of understanding of patients’ perspectives and limitations in study design [26]. There are no data from randomised trials of long-term outcomes following antipsychotic discontinuation among people with recurrent episodes.

The balance between the benefits and harms of long-term antipsychotic treatment requires careful consideration, therefore. Studies that describe people’s experiences of taking antipsychotics, highlight both positive and negative aspects of using medication, and also reveal the complexity of weighing up the pros and cons of drug treatment when making decisions about medication use [11, 13, 27]. Some patients report a lack of involvement in decisions about their treatment and, specifically that requests to reduce or discontinue medication are often not supported [28, 29]. These factors may be related to the low adherence rates amongst patients with schizophrenia [30, 31], which are linked to increased risk of relapse and rehospitalisation [32]. These consequences may result from people stopping medication abruptly without clinical supervision, which may increase withdrawal-related adverse effects including ‘supersensitivity’ psychosis and withdrawal-induced relapse [33, 34]. Despite this, there has been little research that focuses on people’s views about stopping medication. We aimed to explore the views of people with diagnoses of schizophrenia and other psychotic disorders towards taking antipsychotics on a long-term basis, and attitudes towards reducing or stopping these drugs with clinical support. We also aimed to explore the characteristics of people who want to discontinue antipsychotic medication compared to those who do not.

## Methods

### Study design

We conducted face-to-face interviews with people with a diagnosis of schizophrenia spectrum condition using a combination of structured and open-ended questions. The study was also designed to elicit people’s views on taking part in an ongoing randomised trial of supported antipsychotic reduction [35].

### Ethics statement

Ethical approval was provided by the East of Scotland Research Ethics Service (Research Ethics Committee reference: 15/ES/0163).

### Participants

Inclusion criteria consisted of: 1) a clinical diagnosis of schizophrenia, schizoaffective disorder, delusional disorder or other psychotic disorder (excluding bipolar disorder and psychotic depression) as recorded in participants medical notes; 2) a history of either more than one episode of psychosis or schizophrenia, or a single episode that lasted more than a year, 3) taking antipsychotics; 4) being stable for a period of at least 3 months (e.g. not requiring acute care by the crisis team or inpatient unit). Exclusion criteria consisted of; 1) lack of capacity to consent to the research; 2) being legally compelled to take antipsychotic medication; 3) the potential to present a serious risk of harm to self or others in the view of a treating clinician; 4) inability to speak or comprehend spoken English such that an interpreter is required.

### Setting

Participants were recruited from community mental health services and primary care practices across areas of London between April 2016 and August 2017.

In both settings, clinical staff were asked to identify patients meeting eligibility criteria, as far as these could be assessed using clinical records and clinician’s knowledge. Clinical staff then contacted the patients identified either in person or by letter. They explained the study and asked patients if they were willing to be contacted by the research team to receive further information. A researcher then contacted willing participants to arrange a face-to-face interview at a convenient location.

### Data collection

Written informed consent was obtained from all participants prior to conducting the interview. Participants were reimbursed for their time and travel expenses. Trained researchers administered a pre-designed interview schedule, including open and closed questions. The interviews were typically between 30 and 60 min in duration. Participants were given the opportunity to respond to an open question first and then presented with a series of fixed-format, mutually exclusive options and asked to decide (in discussion with the researcher if necessary) which one best captured their views (see Additional file 1 for details of questions).

The schedule was developed based on previous literature and in collaboration with a panel of service users and carers. Participants were also asked to complete the Drug Attitude Inventory (DAI) [36]. The DAI is a 10-item inventory that assesses attitudes towards taking medication for mental health problems and is scored between plus 10 (positive attitude) and − 10 (negative attitude). Parts of the interview including the open questions were audio-recorded with the participant’s consent, and if consent was not given to audio-record, written notes were made.

### Analysis

We conducted quantitative analyses using Statistical Package for Social Sciences (SPSS, Version 25.0) [37]. A pre-planned analysis was conducted to explore factors associated with wanting to stop antipsychotic medication versus not wanting to, or feeling ambivalent. The following plausible explanatory variables were selected and explored using univariate analyses: age, gender, marital status, ethnicity, employment, duration of treatment, antipsychotic dose and form of antipsychotic (depot versus oral). A confirmatory logistic regression analysis was performed.

Qualitative data from open questions were analysed using content analysis in order to identify data that would help clarify responses to quantitative questions, particularly the reasons people gave for their responses. Content analysis is a broad technique that aims to provide ‘a systematic and objective means to make valid inferences from verbal, visual, or written data in order to describe and quantify specific phenomena’ [38]. We used the ‘directed approach’ as set out by Hsieh and Shannon [39] in which knowledge from previous research was used to guide the initial formulation of categories. Data from audio-recordings and written notes were entered into NVivo software (version 11) to facilitate analysis. Initially, seven members of the research team listened to five randomly selected interview recordings to develop an initial set of categories. These were reviewed by the research team, refined, and then a further five interviews were analysed using these refined categories. Categories were subsequently reviewed again and collapsed, divided or further refined in order to produce a set of categories that were conceptually clear, distinct from each other, and were named appropriately to reflect content. These finalized categories were used to analyze the complete data set. Analysis was reviewed for accuracy by two researchers (NC and JT), and the research team met regularly during this process to review uncertainties about how to categorise data, which were resolved by discussion and consensus.

## Results

### Sample characteristics

People were screened for the study from a total of 29 clinical teams across four mental health organisations. Participants were also recruited from 18 primary care practices. A total of 269 patients consented to take part. Characteristics of the sample are reported in Table 1. The mean age of the sample was 46 years (SD = 11.50), 65% (175/269) were male and 52% (137/266) identified as white British or other white background. Most participants were single (67%; 176/264), living alone (66%; 174/262) and unemployed (70%; 187/266).

**Table 1:** Participant characteristics

Characteristics	Total ***N*** = 269 (%)
**Gender N (%)**	
Male	175 (65%)
Female	94 (35%)
**Age in years M (SD) Range**	46.2 (11.50) 21–76
**Diagnosis**^a^ **N (%)**	
Schizophrenia	188 (70.4%)
Schizoaffective Disorder	47 (17.6%)
Delusional Disorder	6 (2.2%)
Drug-induced psychosis	1 (0.4%)
Psychosis/psychotic episodes	17 (6.4%)
Bipolar disorder	5 (1.9%)
Other	3 (1.1%)
**Time in contact with Mental Health Services N (%)**	
< 1 year	2 (0.7%)
1–3 years	14 (5.2%)
4–10 years	67 (25.1%)
11–15 years	45 (16.9%)
16–20 years	44 (16.5%)
> 20 years	95 (35.6%)
**Type of antipsychotic medication N (%)**	
First generation only	82 (30.9%)
Second generation only (excluding clozapine)	128 (48.3%)
Clozapine only	34 (12.8%)
First and second generation (excluding clozapine)	14 (5.3%)
Clozapine plus other antipsychotic	7 (2.6%)
**Services recruited through N (%)**	
Primary Care	41 (15%)
Secondary Care	228 (85%)
**Time taking antipsychotic medication M yrs (SD) Range**	16.5 (10.3) 1–49
**Antipsychotic dose (chlorpromazine equivalent) M (SD) Range**	353.1 mg (269.4) 25–1333 mg
**Number of antipsychotic medications taken N (%)**	
1 antipsychotic	222 (84.1%)
2 or more antipsychotics	42 (15.9%)
**Form of medication N (%)**	
Oral only	131 (49.6%)
Depot only	111 (42.0%)
Both oral and depot	22 (8.3%)
**Drug attitude inventory M (SD) Range**	2.6 (5.1) -8 – 10
**Relationship status N (%)**	
Single	176 (66.7%)
Married/civil partnership/In a long-term relationship	53 (20.1%)
Separated / Divorced / Widow/widower	30 (11.4%)
Other	5 (1.9%)
**Ethnicity N (%)**	
White British/Irish/Other white background	137 (51.5%)
Black or black British	69 (25.9%)
Mixed	15 (5.6%)
Asian or Asian British	36 (13.5%)
Other	9 (3.4%)
**Employment status N (%)**	
Employed	27 (10.2%)
Unemployed	187 (70.3%)
Student	12 (4.5%)
Retired	24 (9.0%)
Voluntary work	16 (6.0%)
**Living situation N (%)**	
Living alone	174 (66.4%)
Living with husband/wife/partner	39 (14.9%)
Living with parents	20 (7.6%)
Living with other relatives/friends/supported living	29 (11.1%)

^a^diagnoses are listed according to those given by participants, which may not have agreed with diagnoses established during screening. Therefore some people are included who described their condition as ‘bipolar disorder’ even though this was not an inclusion diagnosis

Nearly all participants had a diagnosis of either schizophrenia (70%; 188/267) or schizoaffective disorder (18%; 47/267). Most were recruited through secondary mental health services (85%; 228/269), mainly from community mental health teams. Fifteen per cent were recruited through primary care services. The majority of participants had long term involvement with services, with 36% (95/267) of participants reporting being in contact with mental health services for more than 20 years.

The mean length of time taking antipsychotic medications was 16.5 years (SD = 10.31). Most patients (84%; 222/264) were taking only one antipsychotic medication at the time of the interview. In total, 69% (183/265) were taking a second generation antipsychotic (including clozapine), and 50% (133/264) were prescribed a long-acting antipsychotic depot injection.

### Views of continuing, discontinuing or reducing antipsychotic medication

Answers to closed questions revealed that one third (87/265; 33%; 95% CI, 27 to 39) were content to take antipsychotic medication on a long-term basis. A further 19 % (51/265; 95% CI, 15 to 25) accepted it reluctantly. Eighteen percent (47/265; 95% CI, 13 to 23) of participants reported that they were not satisfied taking antipsychotic medication long-term and another 24% (64/265; 95% CI, 19 to 30) accepted taking it for the present but not necessarily forever.

When asked their views about the possibility of discontinuing antipsychotic medication with professional support and supervision, almost a third of patients (82/266; 31%, 95% CI, 25 to 37) reported that they would definitely like to do this, with a further 21% (55/266; 95% CI, 16 to 26) reporting that they had some concerns but would be willing to try. Twenty-one percent (57/266; 95% CI, 17 to 27) wanted to try to discontinue medication in the future, but not at the present moment. Twenty-five percent (66/266; 95% CI, 20 to 31) of patients reported that they did not want to stop their antipsychotic medication.

When asked how they would feel about the possibility of reducing antipsychotic medication with professional support, almost half the participants (118/262; 45%; 95% CI, 39 to 51) reported that they would definitely like to reduce their medication and 13% (35/262; 95% CI, 10 to 18) reported that they would be willing to try to reduce. Fourteen per cent (36/262; 95% CI, 10 to 19) wanted to reduce medication in the future but not at present. Twenty one per cent (59/262; 95% CI, 18 to 28) reported that they would not want to reduce their antipsychotic medication now or in the future.

Table 2 shows the analysis of potential predictors of wanting to discontinue antipsychotic medication. Only DAI score showed a statistically significant association (t(237) = − 8.30; *p* < 0.001), with people with more negative attitudes towards taking medication being more likely to want to discontinue. Notably, no demographic factors, illness or treatment characteristics were associated with wanting to discontinue medication and neither was coming from primary or secondary care services. Multivariable analysis (not shown) confirmed that DAI was the only statistically significant predictor of wanting to discontinue medication.

**Table 2:** Potential predictors of wanting to discontinue antipsychotic medication

Potential predictor variable	% or Mean value (SD) in people who want to stop antipsychotics	% or Mean value (SD) in people who do not want to stop antipsychotics	Mean difference/Odds ratio (95% CI)	*P* value of difference*
Gender	68.6% male (*n* = 94)	61.0% male (*n* = 75)	1.40 (.839–2.33)	.197
Age	45.90 (11.32) (*n* = 135)	46.57 (12.02) (*n* = 122)	−0.67 (−3.54–2.20)	.646
Marital status	73.2% single/unmarried	63.7% single / unmarried	1.55 (.87–2.76)	.173
Ethnicity	50% white British or other	52.2% white British or other	0.92 (.55–1.54)	.846
Employment	85.3% unemployed	83.2% unemployed	0.85 (.40–1.82)	.824
Time taking antipsychotics	14.68 (9.57) (*n* = 65)	17.32 (10.57) (*n* = 142)	−2.64 (−5.67 to .391)	.087
Antipsychotic preparation	44.4% oral only	51.4% oral only	0.76 (.45–1.28)	.366
Dose of antipsychotics (in chlorpromazine equivalents)	328.91 (275.46) (*n* = 64)	365.01 (268.04) (*n* = 152)	−36.10 (−115.48 to 43.27)	.371
DAI total^a^	−.86 (5.16) (*n* = 74)	4.32 (4.11) (*n* = 165)	−5.18 (−6.53 to −3.83)	**<.001**

*p values are derived from t-tests for continuous variables and Chi squared tests for categorical variables; bold indicates significance at the *P* < .05 level

^a^Drug Attitude Inventory

### Content analysis

In total, interviews from 267 participants were analysed including 204 audio recordings and 63 written notes (no notes were made for two of the unrecorded interviews because they were stopped prematurely by the participant). A total of 132 participants provided reasons why they valued or accepted continuing antipsychotic medication (Table 3). The most common reason was fear of relapse, with 70% of the 132 participants citing this. Other common reasons included the view that antipsychotic medication helps to maintain stability or produce a general improvement, that it reduces positive symptoms, particularly hallucinations, the sedative or calming effects of antipsychotics, and the reduction of other symptoms such as agitation and suicidal thoughts. A total of 24% of the 132 participants said that they took antipsychotics because doctors told them to take it.

**Table 3:** Content analysis of participant views about taking antipsychotics on a long-term basis, and attitudes towards reducing or stopping

Category	N (%)^a^	Example or quotes
**Reasons for valuing or accepting long-term antipsychotic medication (*n*** **= 132)**		
Wanting to avoid relapse	93 (70%)	“I relapse much less when I’m on it, so in that sense I’m happy to take it”
General feelings of stability or improvement	48 (36%)	“Helps me stay on an even keel”
Doctor tells me to take them	32 (24%)	“I have to take it because I’m told by my doctors, I have to listen to them, I know that”
Positive symptom reduction	20 (15%)	“The voices are much worse when I don’t take the medication”
Sedative and calming effects	21 (16%)	“Medication keeps me calm and out of trouble”
Indifference, passivity, uncertainty, ambivalence	18 (14%)	“I know that I have to take it, it kind of, doesn’t mean nothing no more, y’know … I just take it”
Other symptom reduction (including depression, agitation, suicidal ideation)	16 (12%)	“My antipsychotic medication is the reason why I’m alive, because I’ve had suicidal thoughts before”
Improved functioning	11 (8%)	“I’m just pleased to be able to function, do normal things”
Other reasons	13 (10%)	E.g. to please family members, habit, to receive welfare benefits, to “not feel different”
**Concerns about long term use of antipsychotics and reasons for wanting to reduce or stop (*n*** **= 121)**		
Unspecified adverse effects	40 (33%)	“Side effects are the main problem … the more you take them, the more you get the side effects”
Sedative effects	36 (30%)	“I feel so relentlessly tired and can’t get out of bed like everyone else”
Weight gain	33 (27%)	“Olanzapine made me put on a huge amount of weight, 3 stones in 3 months without really changing anything”
Neurological effects	31 (26%)	“Makes me weaker, takes my power away. Can’t do as much as I used to, in the gym and things”
Concern about long term health effects	25 (21%)	“I am scared of the unseen damage that it may do to my makings … my chemistry … my makings”
Impact on functioning	20 (17%)	“I seem to function better when I’m not on tablets.. if I wasn’t on the tablets I’d hear more voices, but I had a job, I’d cook, clean, have my own place, when I’m on the tablets I don’t seem to be doing anything”
Dislike the idea of taking long term medication	20 (17%)	“The idea of having to take drugs just to not go crazy doesn’t sit well with me, it makes me feel like I’m not capable of handling life”
Cognitive and emotional side effects	15 (12%)	“Lose your feelings, like you’re a dead person. Want to feel life a bit more”
Doubtful of need for medication	13 (11%)	“I really want to come off it now, because I feel that I am well”
Sexual dysfunction	6 (5%)	“I don’t have interest in sex”
Other adverse effects	5 (4%)	“Sometimes I don’t like it, sometimes it makes me feel a bit bloated and sometimes I get a funny taste in my mouth”
Other reasons	14 (12%)	E.g. Dislike of injections, embarrassment, fear of addiction, inconvenience, doesn’t resolve symptoms, wanting a ‘holistic approach’
**Factors that might facilitate antipsychotic reduction or discontinuation (*n*** **= 61)**		
Support from psychiatrists, other healthcare professionals and services	29 (48%)	“I believe that true collaborative work with the medication staff and myself about reducing my antipsychotic medication is the best way for me to go”
Gradual reduction	15 (25%)	“Not too quick, I don’t think you should drop it too quick. Maybe slowly do it”
Wanting to be independent	10 (16%)	“If I did that [discontinued medication] I’d be on the road to much more independence”
Stable circumstances and healthy lifestyle (e.g. employment, career, diet)	9 (15%)	“I’d need a healthy lifestyle [to reduce]”
Other	10 (16%)	E.g. as required medication, family support, alternative therapies (‘natural remedies’), therapy or counselling.

^a^Responses categories are not mutually exclusive so %‘s do not add up to 100

A total of 121 participants provided data on reasons for wanting to reduce or discontinue antipsychotics. A total of 90 of the 121 participants (74%) cited concerns about the adverse effects of antipsychotic medication and/or its actual and potential impact on their physical health. The most commonly cited specific adverse effects included sedative effects, weight gain and neurological effects (such as shaking, twitching, stiffness, etc). Impairment of general functioning, cognitive and emotional capacities and sexual functioning were also mentioned. Some respondents felt they no longer needed medication, and others simply disliked the idea of taking medication long-term.

A total of 61 participants provided responses that described what they thought would be helpful if they were to consider reducing or discontinuing antipsychotics. Support from psychiatrists and other professionals was most commonly mentioned. Reducing medication gradually and being in a stable situation or having a ‘healthy lifestyle’ at the time of reduction (e.g. being in employment or having a good diet), were considered important by some participants (40%; 24 of 61). Several people (16%; 10 out of 61) reported that the aspiration to be independent or obtain employment was a significant motivation for trying to reduce or stop antipsychotics and might help them to reduce or discontinue medication successfully. Access to a supply of medication to take ‘as required,’ family support, psychological therapy and alternative therapies were mentioned as being potentially useful by some participants (16%; 10 out of 61).

## Discussion

### Main findings

When asked about long-term antipsychotic medication, only a third of participants in this large survey said they were satisfied taking it, whilst others reported that they took it reluctantly, accepted it on a temporary basis or actively disliked it. A third said they would like to try to stop medication with professional support, and almost half wanted the opportunity to reduce medication. Many others were more tentative, but still wanted to try and reduce or discontinue medication, or said they would like to do so in the future. Apart from having more negative attitudes towards medication, which mirrors other research on reasons that people discontinue antipsychotic medication [40], no other characteristics distinguished those who wanted to try and stop medication from others who did not or who felt more ambivalent. The fact that a third of participants were unhappy with the idea of continuing treatment indefinitely is consistent with qualitative research, suggesting that long-term antipsychotic use is often accepted ambivalently as ‘the least worst option’ [11, 13, 29], and with quantitative research that found that 27% of patients with schizophrenia felt their medication had done “more harm than good” [12].

The main reason people valued long-term antipsychotic treatment was for preventing relapse. Other reasons included reduction of positive symptoms, but people also valued antipsychotics for their sedating properties, which contrasts with the official purpose of the medication, but reflects first person accounts reported in qualitative studies [11, 13]. Some people reported taking antipsychotics simply because their doctor told them to. Wanting to stop or reduce medication was motivated by concerns about adverse effects, particularly sedative effects, neurological effects and weight gain, and concerns about antipsychotics’ effects on physical health and functioning, consistent with other research on people’s views about antipsychotic medication [11, 13, 41–43]. Some people felt they no longer needed medication or were curious to know whether they could manage without it, which has also been found in research on reasons for non-adherence [41, 44]. Support from healthcare professionals was identified as being helpful in attempting to reduce or discontinue antipsychotic medication, as was gradual reduction (cited by 25% of patients). This coincides with recent literature on the importance of gradual tapering when reducing or stopping antipsychotic medication to minimise the risk of relapse [45]. Wanting to be independent was regarded as an important motivation for trying to reduce or stop medication.

### Strengths and limitations

With 269 participants, the current study is the largest survey of what people think about continuing or discontinuing antipsychotic medication. The sample was recruited from clinical services, but responses are not necessarily generalizable to the general population of patients who fulfil the eligibility criteria. Although we attempted to make selection as systematic as possible by screening clinical caseloads, inevitably clinical staff influenced who was put forward. This is also likely since the study was linked to a future randomised trial of antipsychotic reduction. People who consent to take part in research may be more adherent and accepting of treatment than other patients [46]; on the other hand, the current study may have appealed to people with more negative views about taking antipsychotic medication. Although not all interviewers were connected to the planned trial, some were, and hence were likely to be committed to the principle that antipsychotic reduction is a viable treatment option. This may have influenced the manner in which questions were presented.

People with long histories of medication use and service contact made up the majority of the sample, which may also have biased responses towards more acceptance of medication. This might also account for the relatively high proportion of patients taking first generation antipsychotics within this study. Therefore, we are not able to produce estimates of the overall rates of different attitudes among our target population, and people with shorter histories are particularly under-represented. The analysis of associations with wanting to discontinue medication could potentially have involved many explanatory variables and been subject to false positive findings. However, the negative association between wanting to discontinue and general attitudes towards antipsychotics is highly plausible.

Content analysis allows a large amount of qualitative data to be converted into numerical form, but categorisation of responses inevitably obscures some of the nuances and complexity of the original responses and does not provide the depth of thematic analysis.

### Interpretation and implications

Qualitative research shows how people who take antipsychotics engage in a sophisticated process of weighing up the pros and cons of treatment based on a variety of considerations including how the drugs influence symptoms and their impact on quality of life and ability to function [13, 29]. In line with previous studies [29], our results suggest that many people take antipsychotic medication reluctantly either because they fear the consequences of stopping or because they accept what their doctor tells them to do. Although the majority do not actively want to change their treatment, a significant minority would like the opportunity to try and reduce or discontinue antipsychotic medication with professional support. They are often motivated by the experience of adverse effects and reasonable concerns about their physical health. Yet, as other research indicates, patients often report that they lack the opportunity to make collaborative decisions about taking antipsychotic medication, and support to try to reduce or discontinue this medication is rarely available [28, 29]. Clinicians can be reluctant to help people reduce or discontinue antipsychotic medication because they are focused on risk, and lack training and official guidance on how best to do this [47]. This is a concern since patients may sense this reluctance, and may reduce or stop medication without sharing this with their psychiatrists [28]. If done abruptly, this may increase the risk of relapse and significant withdrawal effects [14, 33].

Mental health services need to support informed decision-making and patient choice, and to this end services should provide support for patients who wish to try to reduce or discontinue antipsychotics. However, there is a need for guidance on the best approach; current evidence suggests any reduction should be gradual and individualised to each patient to minimize risk of relapse [45, 48]. Routine discussions with long-term patients may reveal people who are dissatisfied with their current treatment for a variety of understandable reasons that are important to explore. Some people who stop medication abruptly and potentially dangerously on their own might cooperate with a planned strategy of supervised reduction conducted in collaboration with their psychiatrist. Yet other patients may wish to switch to a different medication to lessen the impact of specific adverse effects [49].

Further research is required to clarify what patients want and how it might best be offered within mental health services. More research is required on people who have a shorter history, and qualitative work could help to explore patient attitudes in more depth including across different sub-groups (e.g. ethnicity). Identifying the best ways to support people who wish to discontinue antipsychotics is important to alleviate the risks involved. Previous studies of services or novel interventions that aim to minimise antipsychotic use, or encourage greater user choice about medication may provide pointers towards interventions that are evidenced-based and helpful when people are reducing and stopping medication [50–52].

## Conclusion

Many patients diagnosed with schizophrenia or related disorders from community mental health services are unhappy about the idea of taking medication on a continuing or life-long basis, and would like to try reducing or discontinuing their medication at some point. Professional support was identified as important in achieving this. Guidance on how to support people to make informed decisions about long-term antipsychotic treatment, and to reduce or discontinue if they wish, would increase the options that are available to patients who are taking long-term antipsychotics.

## Supplementary Information

**Additional file 1.** Questionnaire contents.

## Data Availability

The data that support the findings of this study are not publicly available due to confidentiality reasons and data protection regulations, but are available from the corresponding author, NC, upon reasonable request.

## Abbreviations

DAI: Drug Attitude Inventory
LEAP: Lived Experience Advisory Panel
NIHR: National Institute for Health Research
RADAR: Research into Antipsychotic Discontinuation and Reduction
SPSS: Statistical Package for Social Sciences
