# Supplementary material for: An analysis of views about supported reduction or discontinuation of antipsychotic treatment among people with schizophrenia and other psychotic disorders
Source: BMC Psychiatry. 2022 Mar 15;22:185. doi: 10.1186/s12888-022-03822-5 (PMC8925064; doi:10.1186/s12888-022-03822-5)
Supplement: Supplementary file 1 — Additional file 1. Questionnaire contents. [file 12888_2022_3822_MOESM1_ESM.pdf]

Additional file 1: Questionnaire contents

Questionnaire content	Questions
Sociodemographic information	1. Age<br>2. Gender<br>3. Marital status<br>4. Ethnic group<br>5. Employment status<br>6. Living situation<br>7. Medication currently taking (i.e. dose, length of time, mode)
Mental health history and medication	1. Mental health diagnosis<br>2. Age when diagnosed<br>3. Length of time in contact with mental health services<br>4. Antipsychotics currently taking (including dose, mode and length of time)<br>5. Length of time taking antipsychotic medication
Antipsychotic medication interview	1. How would or do you feel about taking antipsychotic medication on a long-term basis? [Open text]<br>Select one of the below categories:<br>- I am happy to take antipsychotic medication on a long-term basis<br>- I do not want to take antipsychotic medication on a long-term basis<br>- I am not happy about it, but accept I will have to<br>- I am not sure how I feel about this<br>- I do not want to take antipsychotic medication on a long-term basis but I am happy to take it now<br>- Other<br><br>2. How would you feel about trying to gradually reduce your antipsychotic medication down to a lower dose, if you did this together with your doctor? [Open text]<br>Select one of the below categories:<br>- I would be happy to reduce my antipsychotic medication<br>- I want to stay on the same dose<br>- I might consider this in the future but not now<br>- I would have some concerns about this, but I would be prepared to have a try<br>- I never want to reduce my antipsychotic medication<br>- Other<br><br>3. How would you feel about gradually trying to stop your antipsychotic medication altogether, following a reduction, if you did this with the support of your doctor? [Open text]<br>Select one of the below categories:<br>- I would be happy to stop my antipsychotic medication<br>- I would not want to stop my antipsychotic medication<br>- I would consider this in the future but not now<br>- I never want to stop my antipsychotic medication<br>- I would have some concerns about this, but I would be prepared to have a try<br>- Other
Drug Attitude Inventory (DAI-10)	Views of taking medications and experiences of them [10 items]
